# Supplementary material for: Residue and Risk Assessment of Fluopyram in Carrot Tissues
Source: Molecules. 2022 Aug 29;27(17):5544. doi: 10.3390/molecules27175544 (PMC9457905; doi:10.3390/molecules27175544)
Supplement: Supplementary file 1 [file molecules-27-05544-s001.zip › molecules-1814038-supplementary.pdf]

Table S1. Residues of fluopyram in soil.

Days after Treatment		Untreated Control	Residues ± SD (mg/kg)			
			Root Irrigation		Foliar Spray	
			Treatment at 250.00 g a.i./ha	Treatment at 500.00 g a.i./ha	Treatment at 250.00 g a.i./ha	Treatment at 500.00 g a.i./ha
0	(10 min)	ND	5.34 ± 0.96	14.46 ± 3.95	0.18 ± 0.01	0.33 ± 0.01
	(2 h)	ND	2.43 ± 1.30	17.62 ± 0.74	0.17 ± 0.03	0.59 ± 0.04
	(6 h)	ND	2.50 ± 0.30	6.75 ± 0.99	0.203 ± 0.004	0.62 ± 0.02
1		ND	4.58 ± 0.23	4.13 ± 3.59	0.27 ± 0.01	0.30 ± 0.03
2		ND	3.74 ± 0.66	9.76 ± 0.54	0.21 ± 0.02	0.46 ± 0.04
3		ND	2.14 ± 0.40	8.99 ± 0.51	0.182 ± 0.001	0.31 ± 0.04
5		ND	1.91 ± 0.55	7.15 ± 0.61	0.27 ± 0.01	0.31 ± 0.01
7		ND	0.72 ± 0.04	7.37 ± 0.10	0.51 ± 0.11	1.08 ± 0.02
9		ND	3.93 ± 0.49	11.71 ± 0.94	0.29 ± 0.01	0.57 ± 0.03
11		ND	3.05 ± 0.43	4.28 ± 0.41	0.27 ± 0.02	0.49 ± 0.04
15		ND	5.32 ± 0.16	8.87 ± 3.23	0.18 ± 0.02	0.80 ± 0.03
21		ND	3.32 ± 0.30	5.96 ± 0.81	0.24 ± 0.03	0.52 ± 0.03
28		ND	6.02 ± 0.72	13.83 ± 1.17	0.30 ± 0.02	0.60 ± 0.02

ND = not detected.
